# Pseudorabies virus pUL40 drives inflammatory signaling through competitive hijacking of EphA2 from the Akt–EphA2 interaction

**DOI:** 10.1186/s13567-026-01829-4

**Published:** 2026-08-25

**Authors:** Yutong Tian, Hang Yin, Jiaxiang Fu, Qingqing Yang, Ru Yan, Yuqing Li, Chao Ye, Rendong Fang

**Affiliations:** Joint International Research Laboratory of Animal Health and Animal Food Safety, College of Veterinary Medicine, Southwest University, No.2 Tiansheng Road, Beibei District, Chongqing, 400715 China

**Keywords:** Pseudorabies virus (PRV), Ephrin type-A receptor 2 (EphA2), UL40, Akt, inflammatory responses, phosphorylation

## Abstract

Herpesvirus infection triggers excessive inflammation, contributing to tissue injury and disease severity, but the underlying drivers remain unclear. Here we identify EphA2 as a critical mediator of pseudorabies virus (PRV)-induced inflammatory response. Genetic or pharmacological inhibition of EphA2 reduced TNF-α production in PRV-infected cells and mice, alleviated liver and lung damage, and improved mice survival. Mechanistically, PRV infection promoted EphA2 phosphorylation at Ser-897, which activated NF-κB and MAPK pathways to drive TNF-α production. The PRV tegument protein UL40 directly bound the EphA2 kinase domain (residues 697–901), a region that also interacts with Akt. Unexpectedly, Akt acted as a negative regulator of inflammation, as its knockdown exacerbated cytokine production. UL40 competitively disrupted the constitutive EphA2–Akt complex in a time-dependent manner, relieving Akt-mediated restraint on EphA2 S897 phosphorylation. Accordingly, a UL40-deficient PRV mutant failed to enhance S897 phosphorylation, elicited lower inflammatory responses, and showed attenuated virulence in mice. Collectively, our findings reveal a proviral strategy whereby PRV UL40 hijacks EphA2 to counteract an intrinsic Akt-dependent inhibitory pathway. This UL40–EphA2–Akt cascade is a critical determinant of virus-induced inflammation, and EphA2 S897 represents a potential therapeutic target for mitigating alphaherpesvirus immunopathology.

**Supplementary Information:**

The online version contains supplementary material available at 10.1186/s13567-026-01829-4.

## Introduction

Pseudorabies virus (PRV), also known as suid herpesvirus 1, is the causative agent of Aujeszky’s disease [1], which can infect various mammalian species, significantly impacting the global livestock economy [2, 3]. Furthermore, there have been several cases of human viral encephalitis caused by PRV infection [4]. Due to the close functional relatedness to other herpesviruses, PRV are widely used as model viruses to study viral protein functions [5, 6], and as a live tracer of neuronal circuitry. The mature PRV virion comprises four structural elements—the linear DNA genome, a protective icosahedral capsid, the tegument, and the envelope—and is predicted to encode more than 70 different proteins [7]. Upon PRV infection, the host rapidly mounts an innate immune response, releasing antiviral factors to suppress viral attack [8]. Central to this host defense is the NF-κB signaling pathway, a canonical regulator of inflammatory responses [9]. By modulating the phosphorylation status of diverse downstream effector molecules, NF-κB orchestrates both the initiation and resolution of inflammation during PRV infection, representing a potential target for therapeutic intervention [10].

Ephrin type-A receptor 2 (EphA2) is a single transmembrane protein that composed of an extracellular domain (ECD), a transmembrane segment (TM), and an intracellular domain (ICD) [11]. EphA2, known as a receptor tyrosine kinase, plays critical and diverse roles in biological processes [12]. In tumor research, EphA2 has attracted widespread attention, it can initiate both ligand-dependent tumor-suppressive and ligand-independent oncogenic signaling [13]. This paradoxical behavior is dependent on the manner of activation of the EphA2 receptor [14]. The Eph receptor and its Ephrin ligand were reported to mediate herpesvirus infection. Several herpesviruses, including Epstein–Barr virus (EBV), Kaposi’s sarcoma-associated herpesvirus (KSHV), and human cytomegalovirus (HCMV), exploit EphA2 primarily as an entry receptor by binding to their glycoprotein complexes [15–17]. However, the functions of EphA2 after viral entry, particularly its potential role in modulating host innate immune and inflammatory responses during infection, remain largely unexplored. EphA2 has been reported to be involved in numerous innate immune processes [18, 19]. Given that PRV infection triggers robust innate immune responses and that many viruses target receptor tyrosine kinases to modulate host signaling, we therefore hypothesized that PRV might engage the EphA2 pathway. Here, we report a novel mechanism by which PRV actively manipulates host cell signaling. We found that the PRV tegument protein UL40, a component of the viral ribonucleotide reductase [20], specifically interacts with host EphA2. This interaction triggers the ligand-independent activation of EphA2, enhances its phosphorylation at S897. Mechanistically, UL40 competitively binds to the kinase domain of EphA2, thereby disrupting the constitutive interaction between EphA2 and Akt. We identify Akt as an unexpected negative regulator of PRV-induced inflammation. When UL40 disrupts the EphA2–Akt interaction, this negative regulation is relieved, leading to enhanced inflammatory signaling. Furthermore, we demonstrate that this UL40-driven pS897-EphA2 activation exacerbates the host inflammatory response.

Our study uncovers a previously unknown function of UL40 in viral pathogenesis and identifies it as a key viral factor that hijacks EphA2 to promote a pro-inflammatory state, revealing a novel paradigm in which a viral protein exploits the EphA2–Akt axis to subvert host inflammatory control, hereby advancing our understanding of PRV–host interactions and viral immunomodulation.

## Materials and methods

### Antibodies and reagents

The following antibodies and reagents were used in this study: anti-EphA2 (6997), anti-Phospho-EphA2 (Tyr594) (3970), anti-Phospho-EphA2 (Tyr772) (8244), anti-Phospho-EphA2 (Ser897) (6347), anti-AKT (9272S), anti-IKK beta (8943), anti-Phospho-IKK alpha/beta (Ser176/180) (2697), anti-JNK (4303), anti-Phospho-SAPK/JNK (Thr183/Tyr185) (4668), NF-κB p65 (8242), Phospho-NF-κB p65 (Ser536) (3033), p44/42 MAPK (Erk1/2) (4695) and Phospho-p44/42 MAPK (Erk1) (Tyr204)/(Erk2) (Tyr187) (5726) antibodies were from Cell Signaling Technology; anti-DYKDDDDK tag (20543-1-AP), anti-HA tag polyclonal antibody (51064-2-AP), anti-MYC (16286-1-AP), anti-GST Tag (10000-0-AP), anti-Phospho-EPHA2 (Tyr588) (30263-1-AP) antibodies were from proteintech; anti-Ephrin A1 (A9132) and high-dilution anti-β-actin (AC026) antibodies were from ABclone Technology; 5× sodium dodecyl sulfate (SDS) loading buffer (P0015L), 100× protease inhibitor cocktail for general use (P1005), protein A + G agarose (P2055), GST-tag purification resin (P2251), and NP-40(P0013F) were from Beyotime; ExFect transfection reagent (T101-01) were from Vazyme; NVP-BHG712(HY-13258A) and dimethyl sulfoxide (DMSO; HY-Y0320) were from MCE; 4% paraformaldehyde fixative (BL539A) was from Biosharp; Lipofectamine^™^ 3000 (L3000008) and mouse TNF-alpha enzyme-linked immunosorbent assay (ELISA) Kit (BMS607-3) was from Thermo Scientific; and anti-UL40 antibodies were prepared in-house by our laboratory.

### Plasmid constructs and transfection

The plasmids HA-EphrinA1 and MYC-tagged EphA2-S897A were acquired from BGI (Beijing, China). The plasmids HA-Akt and MYC-EphA2 were purchased from miaolingbio. EphA2 truncation mutants amplified from MYC-EphA2 were inserted into vectors pcDNA3.1 expressing MYC designed as MYC-tagged EphA2 (1–328), EphA2 (21–203), EphA2 (329–529), EphA2 (329–579), EphA2 (580–695), EphA2 (580–977), and EphA2 (697–901), respectively. All primers used for plasmids construction are shown in Additional file 1.

Flag-tagged virus protein plasmids gL was kept in our lab, and flag-tagged virus protein plasmids UL40, UL46, and US1 were kindly gifted by Lei Wu (China Agricultural University, China).

### Cell lines and viruses

All cells including HEK293T, RAW264.7 and Vero were cultured in DMEM (Gbico, USA) supplemented with 10% fetal bovine serum (FBS) and 1% penicillin/streptomycin (Gbico, USA) and maintained at a humidified 37 °C incubator with 5% CO_2_. These cells were infected at a multiplicity of infection (MOI) of 1 for indicated time.

The PRV wild type (PRV-WT) strain was maintained in our lab and the PRV-ΔUL40 strain was kindly provided by Lei Wu (China Agricultural University, China).

### ELISA

Mouse serum and cell supernatant were collected to examine the concentration of TNF-α using Mouse TNF alpha ELISA Kit according to the manufacturer’s instructions.

### Western blotting

At the indicated time points post-infection, both mock-infected and PRV-infected cells were lysed in Radioimmunoprecipitation assay (RIPA) lysis buffer. Protein samples were subjected to SDS-PAGE and subsequently transferred onto a polyvinylidene difluoride (PVDF) membrane by electroblotting. Next, the membranes were blocked with 5% nonfat dry milk and then immunoblotted with indicated primary antibodies (Abs) overnight at 4 °C. Next day, the blots were incubated with horseradish peroxidase-conjugated goat anti-mouse/rabbit IgG. Finally, the distinct protein bands were detected by SuperKine^™^ Universal ECL Substrate (Abbkine Scientific Co., Ltd, China).

### EphA2 knockdown using siRNA interference

Small interfering RNA (SiRNA) was transfected using lipofectamine 3000 (Thermo Fisher Scientific, USA) according to the manufacturer’s instructions. Cells were transfected with 20 μM of EphA2 or Akt siRNA (Sangon Biotech) for 12 h and then infected with PRV as described above. Finally, cell lysates were collected for western blotting. All primers used for siRNA are shown in Additional file 1.

### Generation of EphA2 and EphA2 S897A-overexpressing RAW264.7 cells

For lentiviral packaging, HEK293T cells were co-transfected with MYC-EphA2 (or MYC-EphA2 S897A), psPAX2, and pMD2.G at a ratio of 4:3:1. After 48 h, viral supernatants were collected, clarified by centrifugation at 3000 × *g* for 10 min, and filtered through a 0.45-μm filter. RAW264.7 cells were then transduced with the lentiviral supernatant in the presence of 8 μg/mL polybrene. After 24 h, the medium was replaced with fresh growth medium. Transduced cells were selected with 1.5 μg/mL puromycin for 5–7 days. To obtain monoclonal lines, surviving cells were subjected to limiting dilution in 96-well plates and cultured in puromycin-containing medium for an additional 10–14 days. Single colonies were expanded, and overexpression of EphA2 and EphA2 S897A was confirmed by western blotting using an anti-MYC antibody. Stable lines were maintained in 0.5 μg/mL puromycin.

### Animal experiments

WT C57BL/6 J (total *N* = 117, each group *n* = 3 or 10) and EphA2^−/−^mice (total *N* = 39, each group *n* = 3 or 10) were intraperitoneally infected with 100 μL PRV-WT and PRV-ΔUL40 [5 × 10^5^ 50% tissue culture infectious dose (TCID_50_)] and PBS as blank control. For NVP-BHG712 treatment, mice were pretreated with NVP-BHG712 (10 mg/kg) via oral gavage for 4 days prior to PRV infection. After 72 h infection, mice were sacrificed by cervical vertebrae dislocation and tissues were fixed in 10% formalin and then processed in paraffin for hematoxylin and eosin staining.

### Co-immunoprecipitation (Co-IP) and GST affinity-isolation assays

For Co-IP assay, cells were lysed in NP-40 lysis buffer containing PMSF for 4 h. Then, cell lysates were centrifuged at 12 000 × *g* for 10 min at 4 °C. After centrifugation, supernatants were collected and incubated with indicated Abs followed overnight at 4 °C by the addition of protein A + G agarose for 4 h. After centrifugation at 2500 × *g* for 5 min at 4 °C, the immunocomplexes were washed and then subjected to immunoblotting analysis. Anti-Myc, anti-HA, and anti-Flag Abs were used in this study.

For GST affinity-isolation assay, purified GST (10 μg) and GST-UL40 (10 μg) recombinant proteins were separately mixed with purified MYC-EphA2 (20 μg) in 500 μL NP-40 lysis buffer overnight at 4 °C followed by the addition of GST resin. After centrifugation at 1000 × *g* for 5 min at 4 °C, the pellets were washed with NP-40 lysis buffer and then lysed in RIPA lysis buffer (Beyotime Biotechnology, P0013B) for immunoblotting analysis.

### Protein–protein docking analysis

The structural models for the protein interaction complexes presented in this study were generated through protein–protein docking using GRAMM. The amino acid sequences for the docking calculations were derived from the corresponding UniProtKB entries, including EphA2 (Q03145) and UL40 (G3G964). The protein structures used as templates for docking were obtained from the Protein Data Bank (PDB). The resulting docking complexes were analyzed for protein–protein interactions using the PDBe PISA server and were subsequently visualized using PyMOL.

### Statistics and reproducibility

Data are presented as mean ± standard deviation (SD). For in vitro experiments, data were representative of at least three independent biological replicates with triplicate samples. For in vivo experiments, data were representative of three independent biological replicates with three to ten mice per group. Statistical differences were analyzed using the unpaired Student’s *t*-test for comparisons between two groups and one-way analysis of variance (ANOVA) with an appropriate post hoc test for comparisons among multiple groups. A *P*-value of ≤ 0.05 was considered as statistical significance.

### Ethics statement

Female C57BL/6 J wild-type (WT) mice aged 6–8 weeks were bred in-house or purchased from Byrness Weil biotech Ltd (Chongqing, China). EphA2^−/−^ mice generated via CRISPR/Cas9 technology were kindly provided by Genome Technologies Limited (Chongqing, China). All mice were maintained under specific pathogen-free (SPF) conditions. This study was approved by the Institutional Animal Care and Use Committee (IACUC) of Southwest University, Chongqing, China (IACUC-20231215-02). We have complied with all relevant ethical regulations for animal use.

## Results

### EphA2 promotes PRV-induced inflammatory responses in vitro and in vivo

**Figure 1:**
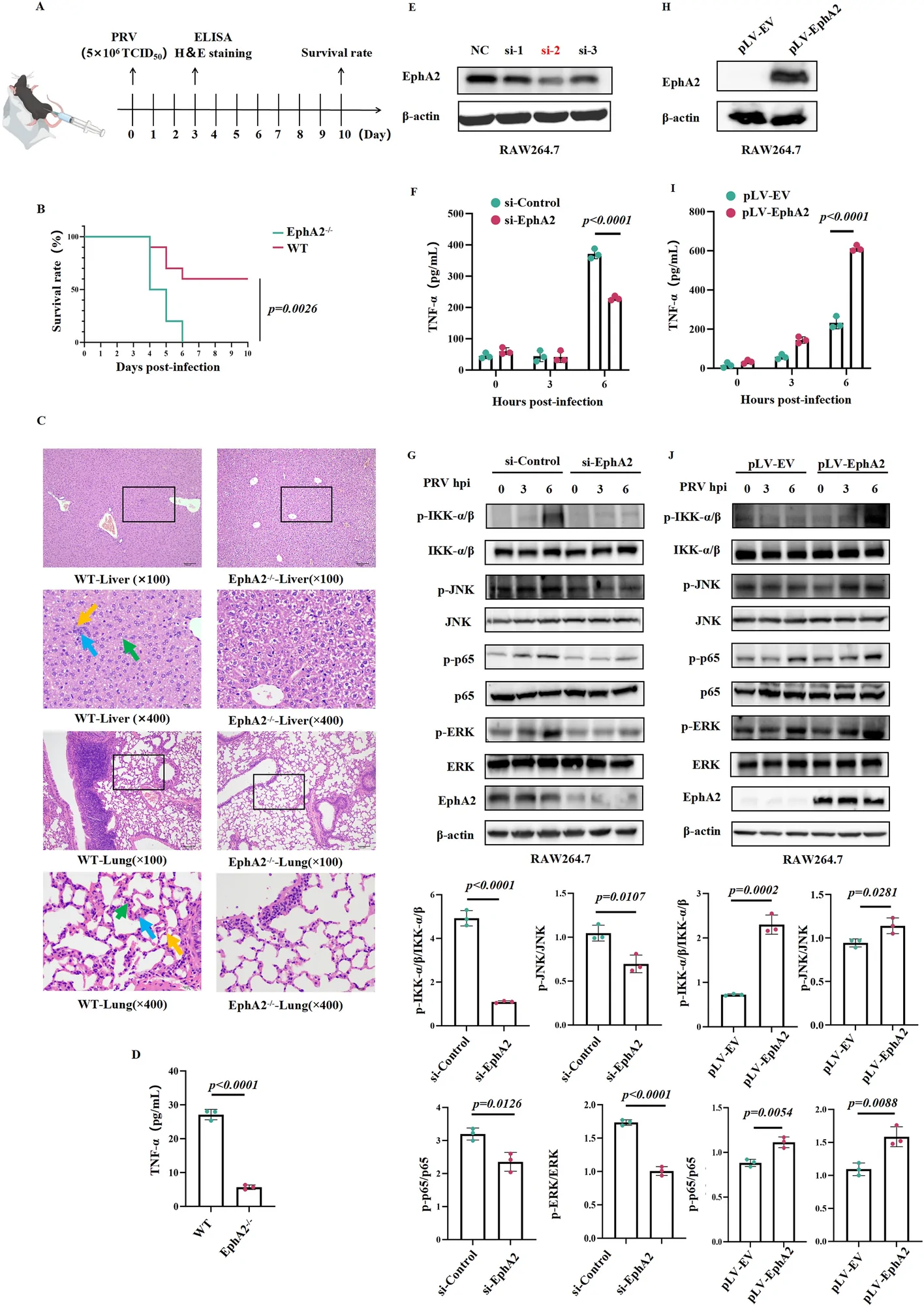
**EphA2 promotes PRV-induced inflammatory responses in vitro and in vivo***.* **A**, **D** The flowchart showing that mice (total *n* = 96, each group *n* = 10 or 3) were infected intraperitoneally by 5 × 10^5^ TCID_50_ PRV. **B** Mice survival rate was recorded for 10 days since infection. After 72 h infection, samples were collected for (**C**) H&E staining and (**D**) ELISA. Inflammatory cell infiltration (green arrows), necrotic cell debris (blue arrows), and reactive gliosis (yellow arrows). **E** The knockdown efficiency of siRNAs targeting EphA2. Three siRNAs that knock down EphA2 were constructed and transfected into RAW264.7 cells, with si-NC used as a negative control. At 24 h post-transfection, the cell lysates were collected to detect the knockdown efficiency via western blotting analysis. **F** Detection of TNF-α in cell supernatant by ELISA. **G** Immunoblot analysis of pERK, ERK, p IKK-α/β, IKK-α/β, pJNK, pJNK, p-p65, and p65 in si-NC or si-EphA2 RAW264.7 cells infected with PRV for indicated time. **H** The overexpression efficiency of EphA2 in RAW264.7 cells. pCMV–MYC-EphA2 were constructed and transfected into RAW264.7 cells, with empty vector used as a negative control. At 48 h post-transfection, the cell lysates were collected to detect the overexpression efficiency via western blotting analysis. **I** Detection of TNF-*α* in cell supernatant by ELISA. **J** Immunoblot analysis of pERK, ERK, p IKK-α/β, IKK-α/β, pJNK, JNK, p-p65 and p65 in RAW264.7 cells overexpressing empty vector or EphA2, following infection with PRV for indicated time points. Data are representative of three independent experiments with triplicate samples in vitro or three or ten mice per group in vivo. All data are presented as mean ± SD. *P* ≤ 0.05 was considered as statistical significance.

EphA2 as a member of the Eph receptor tyrosine kinase family mediates diverse cell signals such as tissue development, cytoskeleton remodeling, endothelial cell barrier integrity, cell adhesion, and migration [21]. Previous studies have demonstrated that EphA2 plays a crucial role in herpesvirus infection [22, 23]; however, its role in PRV-induced pathogenesis remains unclear. We therefore sought to determine whether EphA2 contributes to PRV-induced inflammatory responses in vivo and in vitro. To investigate whether EphA2 is involved in PRV-induced inflammatory response, female wild-type (WT) and EphA2^−/−^ C57BL/6 J mice were injected intraperitoneally (IP) with PRV at a dose of 5 × 10^5^ TCID_50_. Two kinds of mice were each split into three groups: the first group was used for tissue collection on days 3 post-infection to perform hematoxylin and eosin (H&E) staining, the second group was used for serum collection on days 3 post-infection to measure TNF-α levels, and the third group was used for survival monitoring over a 10-day period post-infection (Figure 1A). Consequently, EphA2 deficiency significantly increased the survival rate of PRV-infected mice, coupled with reduced serum TNF-α production and alleviated inflammatory responses in the liver and lungs of infected mice (Figure 1B–D).

To further validate the role of EphA2 in vitro, we knocked down EphA2 using three specific siRNAs in RAW264.7 cells. After examining EphA2 expression by western blotting, we selected the second siRNA for subsequent experiments based on its knockdown efficiency (Figure 1E). In EphA2-knockdown RAW264.7 cells infected with PRV, TNF-α production was significantly reduced compared with control cells (Figure 1F). EphA2 silencing also inhibited the TNF signaling pathway, as evidenced by decreased phosphorylation level of IKK-α/β, JNK, ERK, and NF-κB p65 relative to controls (Figure 1G). In addition, lentivirus-delivered eukaryotic expression vector pCMV–EphA2-MYC-Puro and empty vector were used to overexpression EphA2 in RAW264.7 cells, and the expression of EphA2 was verified by western blot (Figure 1H). Then, EphA2 overexpression significantly increased TNF-α production and enhanced the phosphorylation of the signaling pathway proteins (Figure 1I, J). Collectively, these results demonstrate that EphA2 acts as a positive regulator of PRV-induced inflammatory responses both in vivo and in vitro.

### EphA2 exacerbates PRV-induced inflammation through S897 phosphorylation

**Figure 2:**
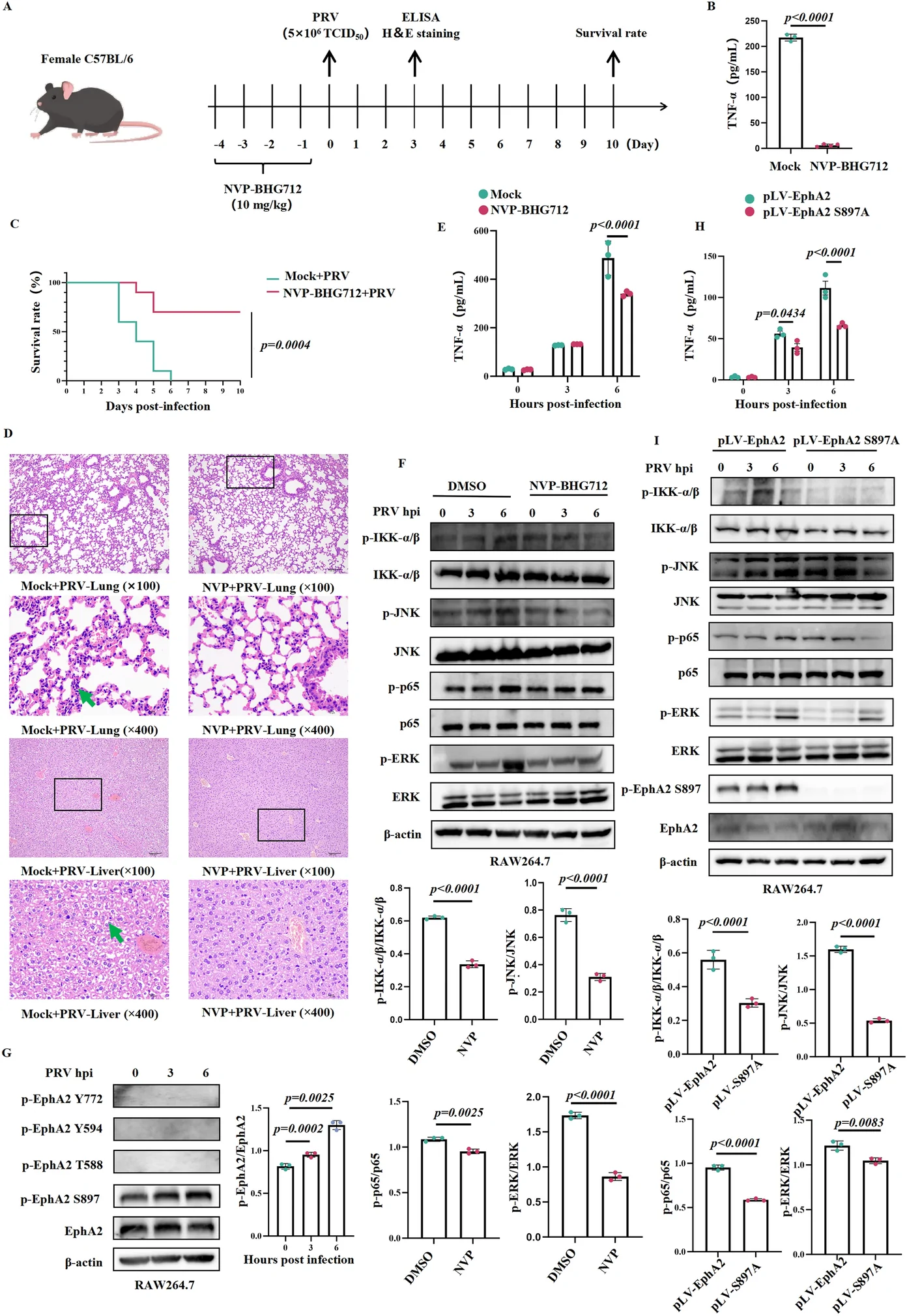
**EphA2 exacerbates PRV-induced inflammation through S897 phosphorylation.** **A**, **D** The flowchart showing that mice (total *N* = 96, each group *n* = 10 or 3) were pretreated with NVP-BHG712 (10 mg/kg) via oral gavage for 4 days prior to 5 × 10^5^ TCID_50_ PRV infection. This figure was created using BioGDP. **B** Mice survival rate was recorded for 10 days since infection. After 72 h infection, samples were collected for (**C**) H&E staining and (**D**) ELISA. Inflammatory cell infiltration (green arrows), necrotic cell debris (blue arrows), and reactive gliosis (yellow arrows). This figure was created using BioGDP. **E** Detection of TNF-α in cell supernatant by ELISA. **F** Immunoblot analysis of pERK, ERK, p IKK-α/β, IKK-α/β, pJNK, pJNK, p-p65 and p65 in RAW264.7 cells pretreated with DMSO or NVP-BHG712 and then infected with PRV for the indicated time points. **G** Immunoblot analysis of EphA2 and its phosphorylation in PRV-infected RAW264.7 cells. **H** Detection of TNF-*α* in cell supernatant by ELISA. **I** Immunoblot analysis of pERK, ERK, p IKK-α/β, IKK-α/β, pJNK, JNK, p-p65, and p65 in RAW264.7 cells overexpressing either wild-type EphA2 or pEphA2 S897A mutant, following infection with PRV for the indicated time points. Data are representative of three independent experiments with triplicate samples in vitro or three or ten mice per group in vivo. All data are presented as mean ± SD. *P* ≤ 0.05 was considered as statistical significance.

The function of EphA2 is tightly regulated by phosphorylation at distinct residues, which differentially control ligand-dependent and ligand-independent signaling [24–26]. We therefore examined whether EphA2 phosphorylation contributed to PRV-induced inflammatory responses. To investigate the role of EphA2 phosphorylation in PRV infection, NVP-BHG712 was used to inhibit nonspecific EphA2 phosphorylation. Prior to injection of viral fluid, female WT C57BL/6 J mice were fed NVP-BHG712 diluted in corn oil for 4 consecutive days (Figure 2A). NVP-BHG712 treatment significantly increased the survival rate of PRV-infected mice, coupled with reduced serum TNF-α production, and alleviated inflammatory responses in the liver and lungs of infected mice (Figure 2B–D). Considering the critical role of EphA2 phosphorylation in vivo, we proceeded to examine its specific effect and mechanisms in vitro. We treated serum-starved RAW264.7 cells with NVP-BHG712 for 4 h, this treatment markedly reduced the inflammatory response in RAW264.7 cells. ELISA results showed that the production of TNF-α were significantly downregulated following NVP-BHG712 treatment (Figure 2E). Moreover, western blot results showed that the phosphorylation levels of IKK-α/β, JNK, NF-κB p65, and ERK in inhibitor-treated cells were also decreased (Figure 2F).

Among multiple phosphorylation sites examined, only Ser-897 phosphorylation was markedly increased following PRV infection (Figure 2G). To directly determine its functional relevance in PRV infection, we generated RAW264.7 cells overexpressing the non-phosphorylatable EphA2 S897A mutant. As a result, blocking Ser-897 phosphorylation significantly reduced TNF-α production compared with that of controls (RAW264.7 cells overexpressing EphA2), the phosphorylation levels of IKK-α/β, JNK, NF-κB p65, and ERK also reduced (Figure 2H, I). Taken together, these findings indicate that PRV-induced inflammatory responses are critically dependent on EphA2 phosphorylation at Ser-897.

### Identification of UL40 as a direct viral ligand binding to EphA2

**Figure 3:**
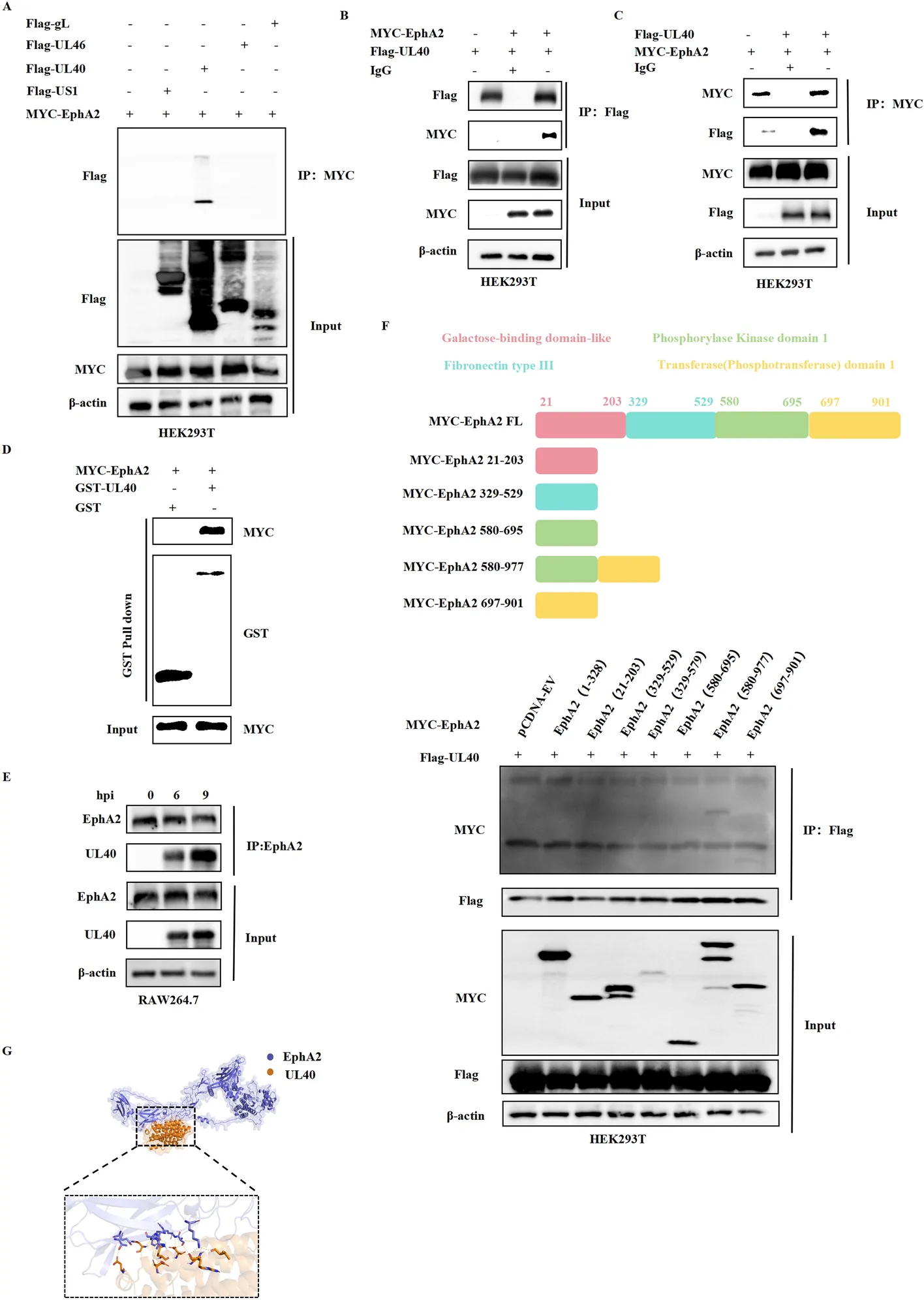
**Identification of UL40 as a direct viral ligand binding to EphA2.** **A** Co-IP analysis of HEK293T cells co-transfected with MYC-EphA2 and viral protein including Flag-gL, Flag-UL46, Flag-UL40, and Flag-US1. **B**, **C** Co-IP analysis of HEK293T cells co-transfected with MYC-EphA2 and Flag-UL40. **D** UL40-EphA2 interaction via GST affinity-isolation assay. **E** Co-IP analysis of the interaction between UL40 and EphA2 in PRV-infected RAW264.7 cells for indicated time. **F** Sketch map of full-length EphA2 along with its truncated mutants. Co-IP analysis of HEK293T cells co-transfected with Flag-UL40, pcDNA empty vector, or EphA2 mutants, including MYC-EphA2(1–328), MYC-EphA2(21–203), MYC-EphA2(329–529), MYC-EphA2(329–579), MYC-EphA2(580–695), MYC-EphA2(580–977), and MYC-EphA2(697–901). **G** Structural model of EphA2-UL40 interaction. Three-dimensional representation of key amino acids involved in the interaction between PRV UL40 (derived from pseudorabies virus) and mouse EphA2 kinase domain (residues 697–901). Yellow dotted lines represent hydrogen bonds. All data are presented as mean ± SD. *P* ≤ 0.05 was considered as statistical significance.

Having demonstrated that PRV-induced inflammatory responses critically depend on EphA2 activation, particularly phosphorylation at Ser-897, we next sought to investigate how PRV infection triggers EphA2 activation. Since receptor tyrosine kinases are commonly activated through direct interactions with viral proteins during infection [27, 28], we sought to determine whether any PRV-encoded protein could physically interact with EphA2 and serve as a viral activator. To identify which PRV protein directly interacts with and activates EphA2, upon reviewing the literature, we found that UL40 and UL46 appear to be intrinsically linked to the virulence of PRV and the inflammatory response it induces [29–31]. We performed a focused co-immunoprecipitation (Co-IP) screen using four viral proteins (gL, UL46, UL40, and US1). These proteins were selected because each has been previously implicated in modulating host signaling pathways: gL facilitates viral entry, UL46 is a tegument protein that affects NF-κB activation, UL40 is predicted to interact with host kinases, and US1 is an immediate-early transcriptional regulator known to associate with host chromatin. HEK293T cells were co-transfected with MYC-EphA2 and individual Flag-tagged viral proteins. Among the candidates tested, only Flag-UL40 showed robust co-immunoprecipitation with EphA2, suggesting a specific interaction (Figure 3A). To further validate this interaction, we performed reciprocal Co-IP experiments. Consistent with the initial finding, Flag-UL40 and MYC-EphA2 co-immunoprecipitated with each other regardless of which protein was used as the bait (Figure 3B, C). To determine whether this interaction is direct, we performed an in vitro GST pull-down assay with purified MYC-EphA2 and GST-UL40. The results demonstrated that GST-UL40, but not the GST control, could specifically capture MYC-EphA2 (Figure 3D), indicating a direct physical association between UL40 and the EphA2 receptor. To further investigate whether UL40 directly regulates EphA2 activity, we performed co-immunoprecipitation (Co-IP) assays in RAW264.7 cells harvested at different time points post-PRV infection. Using an anti-EphA2 antibody to pull down endogenous EphA2, we detected co-precipitated UL40 by western blotting. The amount of UL40 bound to EphA2 increased progressively with infection time (Figure 3E), suggesting that PRV infection promotes the time-dependent interaction between UL40 and EphA2.

We next sought to investigate the key domains of EphA2 interaction with UL40. A series of EphA2 truncation mutants encompassing the galactose-binding domain-like region, fibronectin type III domain, phosphorylase kinase domain 1, and the transferase (phosphotransferase) domain 1 were co-expressed with UL40. Co-IP assays revealed that the deletion mutant lacking the transferase domain 1 failed to interact with UL40, whereas deletions of other regions did not disrupt the interaction (Figure 3F). These findings indicate that the transferase domain 1 of EphA2 is necessary for UL40 binding. To further characterize the molecular interface between EphA2 and UL40, we performed protein–protein docking using HDOCK. The top-ranked docking model exhibited a remarkably favorable binding energy of −5.3 kcal/mol and a docking score of −412.28, indicating a highly stable interaction complex (Figure 3G).

### UL40 disrupts the interaction between Akt and EphA2 by competitive binding

**Figure 4:**
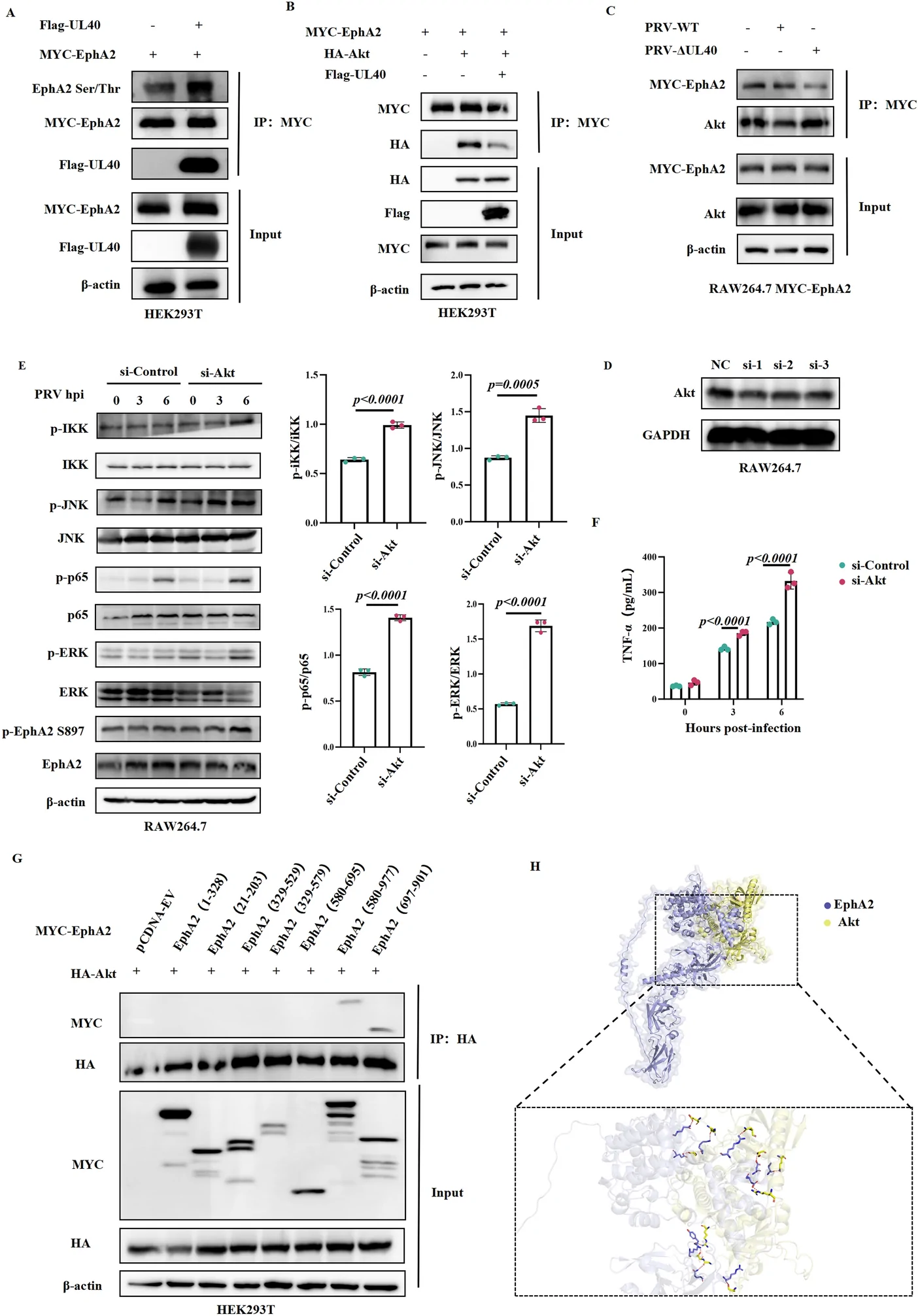
**UL40 disrupts the interaction between Akt and EphA2 by competitive binding.** **A** Co-IP analysis of phosphorylation of EphA2 in HEK293T cells. **B** Co-IP analysis of interaction of Akt and EphA2 in HEK293T cells transfected with MYC-EphA2, HA-Akt in the absence or presence of Flag-UL40. **C** Co-IP analysis of interaction of Akt and EphA2 in MYC-EphA2 RAW264.7 cells infected with PRV-WT or PRV-ΔUL40. **D** The knockdown efficiency of siRNAs targeting Akt. Three siRNAs that knock down Akt were constructed and transfected into RAW264.7 cells, with si-NC used as a negative control. At 24 h post-transfection, the cell lysates were collected to detect the knockdown efficiency via western blotting analysis. **E** Immunoblot analysis of pERK, ERK, p IKK-α/β, IKK-α/β, pJNK, JNK, pp65, and p65 in si-NC or si-Akt RAW264.7 cells infected with PRV for indicated time. **F** Detection of TNF-α in cell supernatant by ELISA. **G** Co-IP analysis of HEK293T cells co-transfected with HA-Akt, pCDNA empty vector or EphA2 mutants, including MYC-EphA2(1–328), MYC-EphA2(21–203), MYC-EphA2(329–529), MYC-EphA2(329–579), MYC-EphA2(580–695), MYC-EphA2(580–977) and MYC-EphA2(697–901). **H** Summary diagram showing that Akt binds to the same EphA2 transferase domain (697‑901) as UL40. **H** Structural model of EphA2–Akt interaction. Three-dimensional representation of key amino acids involved in the interaction between mouse Akt and mouse EphA2 kinase domain (residues 697–901). Yellow dotted lines represent hydrogen bonds.

Having established that UL40 directly interacts with the kinase domain of EphA2 (residues 697–901) and that this interaction is critical for PRV-induced inflammatory signaling, we next sought to investigate how UL40 modulates EphA2 phosphorylation and downstream signaling. Since EphA2 Ser-897 (S897) phosphorylation is a well-established node for its pro-inflammatory function, and Akt is known to be an upstream kinase responsible for this modification [32, 33], we examined whether UL40 influences the phosphorylation status of EphA2. HEK293T cells were co-transfected with EphA2 and UL40, and we observed that UL40 enhanced the serine/threonine phosphorylation of EphA2 (Figure 4A).

To determine whether this enhanced phosphorylation related to Akt, we co-expressed EphA2, Akt, and UL40 in HEK293T cells and performed Co-IP assays. Unexpectedly, the presence of UL40 markedly reduced the interaction between Akt and EphA2 (Figure 4B). This observation was further validated in an independent experimental setting using MYC-tagged EphA2 overexpression in RAW264.7 cells, where co-expression of UL40 similarly attenuated the association between Akt and EphA2 (Figure 4C). These results suggested that, rather than promoting Akt recruitment, UL40 disrupts the constitutive EphA2–Akt complex.

We next sought to understand the functional consequence of this disruption. If Akt binding to EphA2 serves a regulatory function, then modulating Akt expression should influence PRV-induced inflammatory responses. To test this, we knocked down Akt in RAW264.7 macrophages using three independent siRNAs (Figure 4D). Strikingly, upon PRV infection, Akt knockdown led to increased phosphorylation of EphA2 at S897 compared with control cells, with the effect becoming more pronounced over the course of infection. Consistently, Akt knockdown also resulted in elevated TNF-α release (Figure 4F) and enhanced phosphorylation of downstream signaling molecules, including IKK-α/β, JNK, ERK, and p65 (Figure 4E). These findings indicated that, in the context of PRV infection, Akt functions as a negative regulator of inflammatory signaling, likely by restraining EphA2 S897 phosphorylation and its downstream cascade.

To further characterize the molecular basis for the UL40-mediated disruption of the EphA2–Akt interaction, we mapped the binding interface between Akt and EphA2. Using the same series of EphA2 truncation mutants, we performed Co-IP assays with Akt in HEK293T cells. The results revealed that Akt also bound to the transferase domain of EphA2 (residues 697–901) (Figure 4G, H), precisely the same region required for UL40 binding. Given this overlapping binding interface, we reasoned that UL40 may act as a competitive inhibitor, occupying the EphA2 kinase domain and thereby blocking access for Akt.

### PRV-ΔUL40 alleviates PRV-induced inflammatory responses in vitro and in vivo

**Figure 5:**
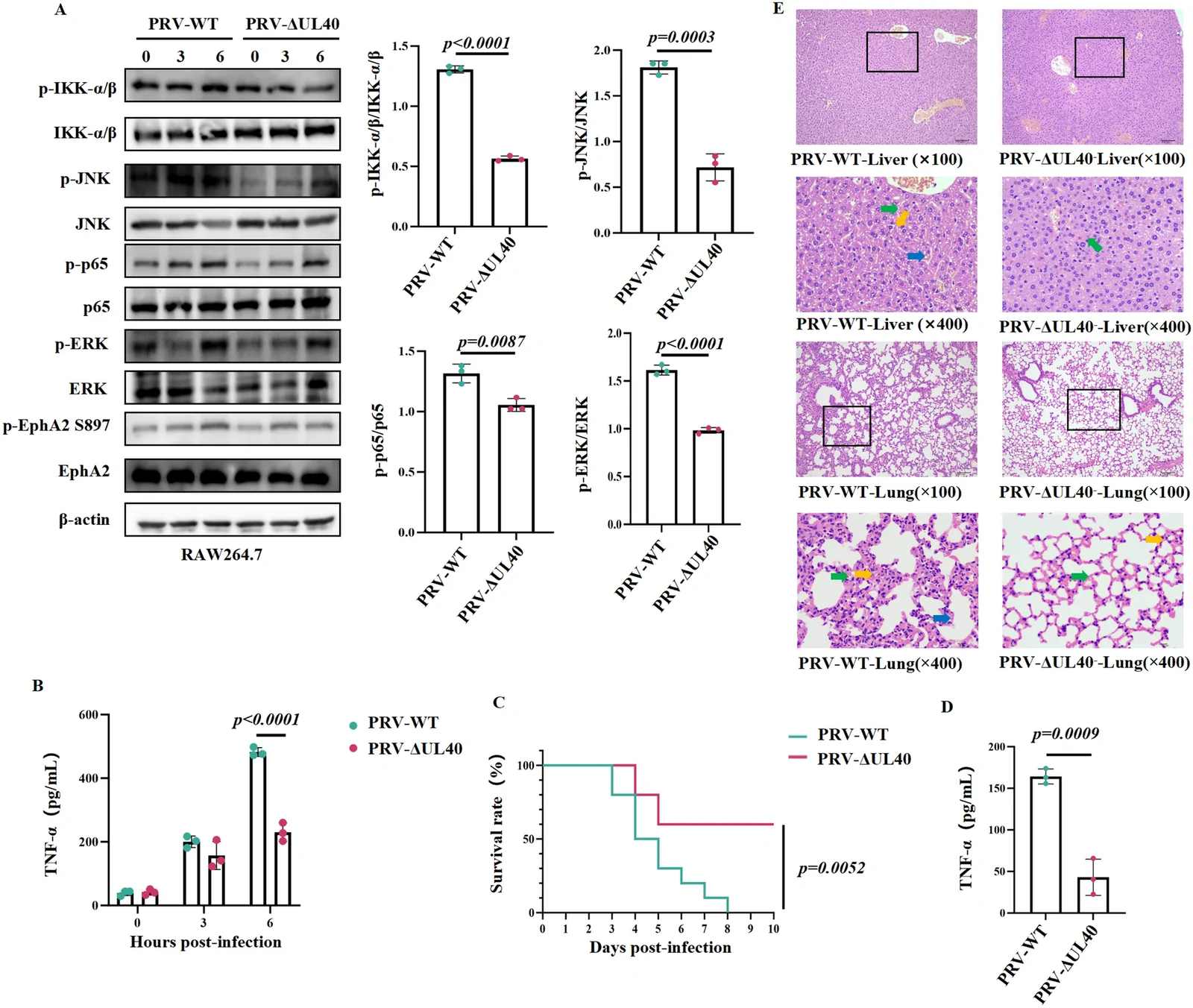
**PRV-ΔUL40 alleviates PRV-induced inflammatory responses in vitro and in vivo***.* **A** Immunoblot analysis of pERK, ERK, p IKK-α/β, IKK-α/β, pJNK, JNK, pp65, p65, pEphA2 S897, and EphA2 in RAW264.7 cells infected with PRV-WT or PRV-ΔUL40 for the indicated time. **B** Detection of TNF-α in cell supernatant by ELISA. **C** Mice survival rate was recorded for 10 days from PRV-WT or PRV-ΔUL40 infection. **D** Detection of TNF-α in mice serum by ELISA. **E** H&E staining of mice with PRV-WT or PRV-ΔUL40 infection. Inflammatory cell infiltration (green arrows), necrotic cell debris (blue arrows), and reactive gliosis (yellow arrows).

To determine whether UL40-mediated EphA2 activation influences host inflammatory responses induced by PRV, RAW264.7 cells were infected with PRV-WT or PRV-ΔUL40 (MOI = 1), activation of the TNF signaling pathway was attenuated in PRV-ΔUL40 infected-cells, as evidenced by decreased phosphorylation of IKK-α/β, JNK, ERK, and p65 (Figure 5A). In addition, PRV-ΔUL40 infection markedly reduced TNF-α production compared with PRV-WT (Figure 5B). To further validate the above phenomena in vivo, female WT C57BL/6 J mice were injected intraperitoneally (IP) with PRV at a dose of 5 × 10^5^ TCID_50_, and they were split into three groups: one for tissue collection for H&E staining on days 3 post-infection, one for serum collection for TNF-α on days 3 post-infection, and one for survival monitoring over 10 days post-infection period. Consistent with the in vitro data, compared with PRV-WT, PRV-ΔUL40 significantly improved survival rates (Figure 5C), reduced serum TNF-α levels (Figure 5D), and alleviated inflammatory injury in the liver and lung (Figure 5E). These findings indicate that UL40 contributes to PRV-induced systemic inflammation and disease severity in vivo. Taken together, these results demonstrate that UL40 acts as a positive regulator of PRV-induced inflammatory responses both in vivo and in vitro by promoting EphA2 phosphorylation activation.

## Discussion

EphA2 has emerged as a multifunctional host factor implicated in tumor biology and viral infection [33]. Among these, EphA2 are increasingly recognized as central hubs exploited by herpesviruses to facilitate entry, replication, and immune modulation [27]. Previous studies have established EphA2 as an entry receptor or signaling cofactor for several herpesviruses, including Kaposi’s sarcoma-associated herpesvirus and Epstein–Barr virus. However, whether EphA2 contributes to inflammatory pathogenesis during PRV infection has remained largely undefined. Our study extends the functional landscape of EphA2 beyond viral entry and identifies it as a key amplifier of inflammatory injury during PRV infection.

In this study, EphA2^−/−^ mice were used to investigate the exact role of EphA2 in PRV induced inflammatory responses. Compared with WT mice, EphA2^−/−^ mice possessed a higher survival rate and reduced TNF-α production, as well as less severe organ damage in the liver and lung following PRV infection, demonstrating the protective role of EphA2 in inducing inflammatory responses during PRV infection.

Pseudorabies (PR) is considered a reemerging infectious disease, consistently threatening the livestock industry in some countries. Epidemics of highly pathogenic PRV variants once caused huge economic losses, which led to great difficulties in the prevention and control of PRV [34]. PRV encodes over 70 proteins, several of which take part in host immunity and evolved several strategies to evade host immunity [35]. Among these viral proteins, the ribonucleotide reductase (RNR) plays an essential role in PRV replication both in vivo and in vitro. This enzyme is a heterotetramer composed of two distinct homodimeric large (RR1/pUL39) and small (RR2/pUL40) subunits. The UL39 has been demonstrated as a potential target for vaccine development and drug design to antagonize viral replication in the infected hosts [20].

EphA2, one of the essential receptor tyrosine kinases (RTKs), plays a significant role in disease progression through its diverse modes of action. It is often overexpressed in cancer, and while ligand-mediated activation tends to inhibit growth and proliferation, overexpression of EphA2 can lead to oncogenic, ligand-independent activation through local aggregation, oligomerization, and autophosphorylation (e.g., T594, T588, Y772, S897, S901)^36^. Notably, Ser-897 phosphorylation represents a well-characterized ligand-independent activation event that promotes cell migration, motility, survival, and proliferation of tumor cells, and is also associated with chemoresistance and drug side effects. In recent years, EphA2 has been identified as a cellular entry receptor for several herpesviruses. It serves as a functional entry receptor for Epstein–Barr virus (EBV) in epithelial cells through direct binding of the viral gH/gL complex to the EphA2 extracellular domain [15]. Similarly, Kaposi’s sarcoma-associated herpesvirus (KSHV) engages EphA2 via its gH/gL glycoprotein complex, and this interaction can be blocked by small-molecule inhibitors targeting the EphA2–ephrin binding interface [28]. Structural studies have further revealed the molecular basis of EphA2 recognition by γ-herpesviruses gHgL complexes, suggesting that multiple animal *γ*-herpesviruses may exploit EphA2 as an entry receptor [27]. For human cytomegalovirus (HCMV), EphA2 has also been implicated as a functional entry receptor, and its role in maintaining virus latency through cooperation with viral proteins has been speculated [17]. Beyond herpesviruses, EphA2 has been linked to rhinovirus-induced innate immune responses in sinonasal epithelial cells and to Seneca Valley virus replication. However, the role of EphA2 in viral inflammatory pathogenesis has remained largely unexplored. In this work, we identify a novel mechanism by which pseudorabies virus (PRV) exploits EphA2 to drive inflammatory injury. Our key finding is that the viral protein UL40 directly binds to the kinase domain of EphA2 (residues 697–901) and functions as a competitive ligand. Specifically, UL40 engages the same region within EphA2 that mediates its constitutive interaction with the serine/threonine kinase Akt. By occupying this domain, UL40 displaces Akt from the EphA2–Akt complex, thereby relieving Akt-mediated negative regulation. This displacement consequently enhances EphA2 S897 phosphorylation and triggers downstream inflammatory signaling, including increased TNF-α production and activation of IKK-α/β, JNK, ERK, and p65 pathways. Thus, UL40 acts as a molecular switch that converts EphA2 from a restrained state to a pro-inflammatory amplifier. Interestingly, while Akt has traditionally been viewed as a pro-survival and pro-inflammatory kinase in many contexts [36, 37], our study reveals an unexpected, context-dependent role for Akt during PRV infection. We found that Akt constitutively interacts with the EphA2 kinase domain (residues 580–977) and functions as a negative regulator of PRV-induced inflammation. Knockdown of Akt led to enhanced EphA2 S897 phosphorylation, increased TNF-α production, and augmented activation of downstream signaling cascades.

Furthermore, we demonstrate that PRV activates a noncanonical, ligand-independent EphA2 signaling program. In physiological contexts, EphA2 is typically activated by binding to ephrin-A ligands, resulting in tyrosine autophosphorylation and receptor internalization. In contrast, ligand-independent signaling (phosphorylation at Ser-897) has been primarily studied in cancer, where it promotes sustained MAPK and NF-κB activation. This observation expands the biological relevance of ligand-independent EphA2 signaling from tumor progression to viral immunopathology. Another our contribution is the identification of UL40 as a direct viral ligand that engages EphA2 intracellularly. Mechanistically, we demonstrate that UL40 competitively binds to the transferase domain of EphA2 (697–901), the same region that mediates interaction with Akt. By occupying this domain, UL40 disrupts the constitutive EphA2–Akt complex, thereby relieving Akt-mediated negative regulation and promoting S897 phosphorylation and downstream inflammatory signaling [38]. This competitive occupancy model provides a mechanistic explanation for our observations: UL40 binds to EphA2 (697–901), displaces Akt from the complex, relieves Akt-mediated negative regulation, and consequently promotes EphA2 S897 phosphorylation and downstream inflammatory signaling. Taken together, these data uncover a mechanism by which PRV UL40 promotes inflammation: UL40 competitively binds to the kinase domain of EphA2, disrupting its interaction with Akt, which normally functions to restrain EphA2 S897 phosphorylation and downstream inflammatory responses.

In summary, we uncover a mechanism by which PRV exacerbates inflammatory responses via the UL40–EphA2–Akt axis. UL40 acts as a molecular competitor that displaces Akt from the EphA2 kinase domain, relieving Akt-mediated inhibition and promoting EphA2 S897 phosphorylation and downstream inflammatory signaling. This work redefines EphA2 beyond its role in herpesvirus entry, establishing it as a key amplifier of virus-induced immunopathology and revealing a novel proviral strategy that subverts an intrinsic host checkpoint to drive inflammation.

## Supplementary Information

**Additional file 1**. **All primer sequence information mentioned in this article**.

## Data Availability

No datasets were generated or analyzed during the current study.
