# Supplementary material for: Pseudorabies virus pUL40 drives inflammatory signaling through competitive hijacking of EphA2 from the Akt–EphA2 interaction
Source: Vet Res. 2026 Aug 25;57:153. doi: 10.1186/s13567-026-01829-4 (PMC13508209; doi:10.1186/s13567-026-01829-4)
Supplement: Supplementary file 1 [file 13567_2026_1829_MOESM1_ESM.docx]

**Supplementary Table 1.** All primer sequence information mentioned in this article

**siRNAs sequences**

| **Name of primer** | **Base sequence (5' to3')** |
| --- | --- |
| m*EphA2-*147 | GGUGGGACCUGAUGCAGAA |
| m*EphA2*-189 | ACAUGUACUCGGUGUGCAA |
| m*EphA2*-342 | AAGAGACCUUCAACCUCUA |
| m*Akt*-491 | UGAUUCUGGUGAAAGAGAA |
| m*Akt*-872 | CGGACUUCGGGCUGUGCAA |
| m*Akt*-1149 | GCUCAAGAAGGACCCUACA |

**Primer sequences for plasmids**

| **Name of primer** | **Base sequence (5' to3')** |
| --- | --- |
| pCDNA3.1-MYC-*EphA2*(1-328)-F-BmaHI | TTGGTACCGAGCTCGGATCCATGGAGCTCCGGGCAGTCG |
| pCDNA3.1-MYC- *EphA2* (1-328)-R-EcoRI | GATATTAGTTTTTGTTCCTCGAGACGTGTGCAAGACATGGACA |
| pCDNA3.1-MYC- *EphA2* (21-203)-F-BmaHI | GTCTGCAGCTAGCCACCATGGCGGCGGCACAGGGAAAGGAA |
| pCDNA3.1-MYC- *EphA2* (21-203)-R- EcoRI | TATTAGTTTTTGTTCCTCGAGCATCTCGGGACACTTCTTGTAG |
| pCDNA3.1-MYC- *EphA2* (329-529)-F-BmaHI | GTACCGAGCTCGGATCCATGCCACCCTCTGCCCCCAACTA |
| pCDNA3.1-MYC- *EphA2* (329-529)-R- EcoRI | TATTAGTTTTTGTTCCTCGAGGGACAGTGTCTGGAACTCGTG |
| pCDNA3.1-MYC- *EphA2* (329-579)-F-BmaHI | GTACCGAGCTCGGATCCATGCCACCCTCTGCCCCCAACTA |
| pCDNA3.1-MYC- *EphA2* (329-579)-R- EcoRI | ATTAGTTTTTGTTCCTCGAGCTTGGAAAAACGGACATCCTC |
| pCDNA3.1-MYC- *EphA2* (580-695)-F- BmaHI | GTACCGAGCTCGGATCCATGTCAGAACAACTAAAGCCCCT |
| pCDNA3.1-MYC- *EphA2* (580-695)-R- EcoRI | TTAGTTTTTGTTCCTCGAGGTACTCTGTGATAATCATCATG |
| pCDNA3.1-MYC- *EphA2* (580-977)-F- BmaHI | GTACCGAGCTCGGATCCATGTCAGAACAACTAAAGCCCCT |
| pCDNA3.1-MYC*-EphA2* (580-977)-R- EcoRI | GATATTAGTTTTTGTTCCTCGAGGATAGGAATCCCCACTGTGT |
| pCDNA3.1-MYC- *EphA2* (597-901)-F- BmaHI | TACCGAGCTCGGATCCATGGAGAATGGAGCGCTAGACAAG |
| pCDNA3.1-MYC- *EphA2* (597-901)-R- EcoRI | TATTAGTTTTTGTTCCTCGAGGCCGCTGGTGCTGGGCAGCC |
| pGEX-GST-*UL40*-F-BamH1 | ATCTGGTTCCGCGTGGATCCATGGAGTACTTTTACAC |
| pGEX-GST-*UL40*-R-XhoI | ACGATGCGGCCGCTCGAGTCATCGTCGTCCTTG |
